# Evolution and Contribution of a Global Partnership against Measles and Rubella, 2001–2023

**DOI:** 10.3390/vaccines12060693

**Published:** 2024-06-20

**Authors:** Peter Strebel, Mark Grabowsky, Edward Hoekstra, Andrea Gay, Stephen Cochi

**Affiliations:** 1 Independent Consultant, 1290 Geneva, Switzerland; 2 Independent Consultant, Oxford, CT 06478, USA; 3 Independent Consultant, New York, NY 11375, USA; 4 Washington, DC 20012, USA; 5 Atlanta, GA 30345, USA

**Keywords:** Global health partnerships, Epidemiology of measles, Epidemiology of rubella

## Abstract

This article describes the arc of global measles and rubella elimination since 2000 from the perspective of the founding partners of the Measles Initiative. The Measles Initiative was formed in 2001 as a partnership among the American Red Cross, the Centers for Disease Control and Prevention, UNICEF, the United Nations Foundation, and the World Health Organization with the aim to reduce measles deaths in low-income countries. Recognizing rubella as the leading infectious disease cause of congenital abnormalities globally and achievement of measles and rubella elimination in the region of the Americas, the partnership was renamed the Measles and Rubella Initiative (MRI) in 2012. The goals of the MRI were at least a 95% reduction in global measles mortality and elimination of measles and rubella in at least five of the six WHO regions. In January 2023, the membership of the partnership was expanded to include the Bill and Melinda Gates Foundation (BMGF) and Gavi the Vaccine Alliance, and its name changed to the IA2030 Measles and Rubella Partnership. We describe the role the partnership has had in measles partner effectiveness and its impact on measles and rubella disease burden, including how the partnership has strategically adapted to the evolving immunization landscape. We conclude with lessons learned regarding the role global partnerships can play in furthering the impact of disease control programs within the current global immunization environment.

## 1. The Measles Initiative—The First 10 Years, 2001–2010

Despite substantial progress in the Americas during the 1990s, measles remained the leading cause of vaccine-preventable deaths worldwide, with more than ¾ of a million deaths in 1999 [1]. Ninety-four percent of measles-associated deaths occurred in 47 countries, mainly in Africa [1]. In the late 1990s, seven countries in southern Africa successfully applied PAHO’s strategy featuring nationwide mass measles vaccination campaigns, which were country financed and reduced the number of measles-related deaths to near zero in those geographies [2]. Based on those demonstrated successes, other endemic countries were eager to do the same; however, they lacked the resources and technical support to move forward. The Measles Initiative was formed in 2001, with each of the five founding organizations bringing a different perspective and capacity to address these needs.

In its first decade, the Measles Initiative worked with 70 countries, offering financial and technical support to vaccinate more than 900 million children in supplementary immunization activities at a cost of less than USD 1 per child vaccinated [3]. In 2003, with evidence from rapid success in Africa, the World Health Assembly committed to reducing global measles deaths by 50% between 1999 and 2005. Countries outside of Africa expressed interest in mass vaccination strategies, and the Measles Initiative began to support governments worldwide the following year. Not only did the Measles Initiative support vaccination efforts but it also began to support a laboratory network and measles disease surveillance. By the end of 2005, the Measles Initiative, through the committed efforts of national governments, had catalyzed a reduction in global measles deaths by 60% relative to 1999, thereby exceeding the United Nations’ target [1]. To continue the momentum, the World Health Assembly endorsed a more ambitious goal—to reduce measles deaths by 90% between 2000 and 2010. By the end of 2008, 2 years ahead of schedule, all countries but one (India) had reached the 2010 goal. Global measles deaths have been reduced by 78% [3]. Between 2000 and 2010, first-dose coverage (Measles vaccination strategies include vaccination coverage of ≥95% with the first and second dose of measles vaccine supplemented by wide age range campaigns to vaccinate children who have not had two doses of vaccine) rose from 72% to 84%, and second-dose coverage increased from 17% to 42% [4]. In addition, measles campaigns were successful in delivering more than 41 million insecticide-treated nets, 94 million deworming tablets, 127 million polio vaccines, and 213 million doses of vitamin A [3].

The Measles Initiative initially had an informal but well-coordinated and focused management style. The goal was to provide much-needed technical and financial support to national measles plans, immunization campaigns, and measles surveillance while securing as much national buy in and ownership as possible. Plans were developed at the local and national levels. To supplement national commitment, external funding decisions were made collectively by representatives from each partner in the Measles Initiative. The Measles Initiative functioned without a formal secretariat. As a result, each organization maintained its own internal information systems, and there was no common data repository. The responsibility for managing and coordinating activities was shared equally. Partners jointly mobilized resources and pooled their funds for national program implementation, requiring only a plan of action and report on the activities funded by the Measles Initiative from each country. Each country’s single plan and budget prevented parallel funding, ensured joint planning at the country level, and enabled flexibility. This structure streamlined program planning and implementation by lowering transaction costs for governments and partners. New partners at the local level were encouraged. The partnership was inclusive and supported local efforts to improve national immunization programs.

The ensuing rapid expansion, increasing visibility, and growing financial requirements put significant stress on the management of the Measles Initiative. Although the partnership initially experienced a several years-long period of evolutionary continuous growth without a significant setback or internal disruption, after its first decade, the Measles Initiative experienced a period of challenges that required “revolutionary” changes.

## 2. Consolidation of Progress Leads to Increasing Challenges (2011–2020)

The remarkable progress toward measles and rubella elimination during the first decade of the Measles Initiative resulted in heightened political, technical, and programmatic support to pursue measles eradication as the decade of the 2010s began [5]. Recognizing rubella as the leading infectious cause of congenital abnormalities globally, the Measles Initiative was renamed the Measles and Rubella Initiative (MRI) in 2012. In the Americas, the last cases of endemic measles transmission occurred in 2002, and the last cases of rubella and congenital rubella syndrome (CRS) in 2009 [6]. An expert advisory panel convened by the WHO in 2010 concluded that measles can and should be eradicated, and the WHO Strategic Advisory Group of Experts (SAGE) on Immunization endorsed these conclusions. In 2011, the WHO’s Executive Board endorsed the SAGE recommendations [5,7]. By 2013, member states of all six WHO regions had voted for regional measles elimination target dates on or before 2020, securing the political commitment of all countries. Countries and regions began pursuing accelerated rubella elimination following the successful verification of rubella elimination in the region of the Americas, which has been sustained until now [5]. Progress in rubella-containing vaccine (RCV) introductions was facilitated by a policy recommendation for the use of a combined measles–rubella (MR) vaccine [8] and the Gavi commitment of USD 500 million for the rubella vaccine introduction that began in 2012. All six WHO regions established regional measles and rubella elimination verification commissions to monitor progress. By mid-decade, the WHO regions and MRI-supported countries were encouraged by the momentum that had been achieved.

Programmatic improvements matched these political and technical commitments. The number of countries providing a second dose of measles vaccine (MCV2) increased markedly from 98 (51%) in 2000 to 182 (94%) in 2020, and the estimated global MCV2 coverage increased from 17% in 2000 to 72% in 2020 [4]. The number of countries providing rubella vaccine increased from 130 (67%) in 2010 to 173 (89%) countries in 2020, and estimated global coverage increased to 70%, resulting in a two-thirds decline in estimated congenital rubella syndrome (CRS) births from 100,000 in 2010 to 32,000 in 2019 and averting an additional 229,000 CRS cases [9]. The introduction and expanded use of RCV in all regions since 1970 averted an estimated 696,859 CRS births globally during 2010–2019 when compared to the situation that might have been expected if RCV had not been introduced anywhere (written communication, Emilia Vynnycky, 30 January 2024)*.* As of March 2024, 19 countries have yet to reduce RCV in their national immunization programs. By 2016, measles incidence and deaths reached record lows compared with rates at the inception of the partnership in 2001. Annual reported measles incidence had decreased by 87%, from 145 to 19 cases per million persons, and annual estimated measles deaths fell by 84% to a record low of 89,780; between 2000 and 2016, measles vaccination prevented an estimated 20.4 million deaths [10].

However, there were clouds on the horizon. Global-level political commitment and momentum for measles and rubella elimination began to dissipate by the middle of the second decade. There was a gradual decline through the end of the decade, despite sustained commitment at the regional level. Impeding factors included (1) a global inability to achieve polio eradication, resulting in disenchantment with eradication initiatives and concerns as to the practical feasibility and true costs of achieving measles eradication; (2) a parallel shift away from vertical health programs; (3) the consolidation of the flow of immunization funding toward Gavi at the expense of disease-specific initiatives; and (4) discontent with the need for, and expense of, frequent mass campaigns (themselves of variable quality) because routine immunization programs were not mature enough to sustain high levels of measles immunity.

Although the strategic approaches to measles and rubella elimination continue to remain valid, they were not fully implemented, mainly due to a lack of global political will, as reflected in inadequate resources and, in some cases, a lack of country ownership [11]. Average annual funding during 2001–2016 was USD 69 million for the MRI, in contrast to USD 818 million for the Global Polio Eradication Initiative [7]. As part of a more comprehensive measles–rubella strategy, Gavi funding increased to USD 820 million (i.e., average annual funding of USD 164 million) for measles–rubella control activities for the strategic period 2016–2020 [12]. However, some key donors expressed reservations about committing additional resources to eliminate measles and rubella. These partners were concerned that committing additional resources to achieve elimination targets in countries that rely on donor support for campaign operational costs would promote mass campaigns over routine service delivery. Subsequently, a worldwide measles resurgence occurred during 2018–2019, the root cause of which was a failure to vaccinate young children, particularly in marginalized communities, as well as school-aged children and young adults facilitated by international spread [13]. The final blow to further progress was the arrival of the COVID-19 pandemic in 2020 with its associated growth in anti-vaccine sentiment and substantial disruption of all immunization services and disease surveillance.

Frustrated by the slow progress toward elimination in other regions and the persistent risk of measles importations, member states of PAHO pushed for establishing a measles eradication goal at the 2017 WHA. A compromise was reached calling for a feasibility assessment of measles and rubella eradication to be presented to the WHA in 2020. Based on a comprehensive review, the feasibility assessment report concluded that while eradicating both measles and rubella was biologically and technically feasible, public, political, and donor support was lacking [14]. Rather than pursue a top-down approach to eradication, the report recommended that the path to measles and rubella eradication should strengthen primary health care and be a pathfinder for the more bottom-up integrated approach laid out in the Immunization Agenda 2030 (IA2030). The conditions for establishing a time-bound measles and rubella eradication goal included setting up a monitoring and accountability framework and achieving predetermined benchmarks, as well as a short and financially feasible end-game strategy.

## 3. Adapting to a Changed Global Immunization Environment (2021 to 2023)

In response to the need for alignment across the global immunization strategy for the coming decade, the MRI partners developed the new Measles and Rubella Strategic Framework 2021–2030 (MRSF2030) with a vision of “A world without measles and rubella” and the goal of achieving and maintaining regional measles and rubella elimination goals [15]. The MRSF2030 called for embedding measles and rubella activities within country immunization and Primary Health Care programs (a key lesson learned from the PAHO approach), a better definition of the roles and responsibilities of stakeholders at each level, strengthening country capacity for outbreak response, and expediting innovative vaccine delivery technologies, such as micro-array patches and rapid diagnostic tests to bolster surveillance. However, the MRSF stopped short of providing an implementation roadmap or a monitoring/accountability framework.

In 2020, the MRI Management Team commissioned a thorough assessment of the global measles and rubella partnership, including recommendations on what would be needed to deliver on the promise of the MRSF2030. The report highlighted the rapid evolution in the global immunization ecosystem characterized by competing country priorities, demand for delivery integration, the growing influence of BMGF and Gavi, and widescale disruption in health care and immunization services due to the COVID-19 pandemic.

To be successful in this new reality, the report recognized that a refreshed partnership would need a sharp strategic focus and more efficient ways of working. Recommendations include (1) integrate the MRI with IA2030 to reap the benefits of linkages with other partners and horizontal approaches; (2) strengthen partnership management by allowing for broader membership in the management team; (3) create a centralized support unit to drive progress and implement new ways of working; (4) commit to monitoring program milestones with regular “stock takes” and take corrective actions; and (5) determine which partnership functions should be incorporated into emerging IA2030 structures.

In the face of a post-pandemic resurgence of measles, implementing key recommendations from the Partnership Assessment has been expedited. As of January 2023, BMGF and Gavi have become full members of the newly renamed IA2030 Measles and Rubella Partnership [16]. The partnership has also created a secretariat to support its strategic activities and goals. However, the IA2030 infrastructure is still under development, and much work remains to effectively integrate the remaining shared functions and adopt more efficient and effective new ways of working.

## 4. Overall Partnership Impact and Lessons Learned

While most partnership support has been directed toward mass vaccination campaigns and improving measles and rubella surveillance, it has also contributed to improvements in routine immunization coverage. Between 2000 and 2022, global MCV1 coverage increased from 72% to 83%, and MCV2 coverage from 17% to 74% [4]. This four-fold increase in MCV2 coverage was driven by the introduction of a routine second dose in ninety-three countries, most of which benefited from Gavi funding that began in 2007. MCV2 still needs to be introduced in six countries. Support from the MR partnership also significantly impacted global RCV coverage, which has increased from 40% in 2012 to 70% in 2020 [9]. This increase was primarily due to the introduction of RCV in countries in the Southeast Asian and Western Pacific Regions. RCV remains to be introduced in 19 countries, mainly in the African Region, and its introduction is being impeded by a lack of agreement on whether RCV can be safely introduced in countries with very weak immunization services [8]. Results from a modeling study by Frey et.al. published in this supplement suggest that this concern may be unfounded.

Over the course of the partnership, reported measles incidence has decreased by 80% from 145 cases per million population in 2000 to 29 in 2022. Similarly, estimated measles deaths have decreased by four-fifths (82%), from 773,000 in 2000 to 136,000 in 2022, with 57 million (recent updates to the mathematical model that include more dynamic approaches to estimating the age distribution of measles cases, as well as age-specific measles case–fatality ratios [17], have shifted cases to younger age groups with higher case-fatality ratios. This resulted in higher overall estimates of the cumulative number of measles deaths averted when compared to estimates that were based on earlier models) cumulative measles deaths averted by vaccination [4]. Global CRS births have declined by more than two-thirds since 2010, from 100,000 in 2010 to 32,000 in 2019 [9], and the use of RCV since 1970 averted an estimated 696,859 CRS births globally during 2010–2019 (written communication, Emilia Vynnycky, 30 January 2024).

Technical and financial support from the partnership has enabled the expansion of the Global Measles and Rubella Laboratory Network (GMRLN) to include 734 national and subnational WHO-certified laboratories, a considerable increase from fewer than 40 laboratories in 1998. The number of measles genotypes detected by GMRLN decreased from thirteen in 2002 to six in 2014, three in 2020, and two (D8 and B3) in 2022, reflecting the increasing pressure of measles vaccination on measles virus transmission and genotype diversity [4].

The first decade of the partnership saw rapid progress due to the rolling out of PAHO measles elimination strategies in Africa and other WHO regions [5,6]. The game-changing approach was conducting catch-up campaigns that targeted multiple cohorts of children and included the vast majority of measles-susceptible individuals (in this context, ages 9 months to 14 years), leading to rapid increases in population immunity. Marked drops in measles incidence and a more than three-quarters reduction in global measles mortality resulted, generating optimism that measles could be eradicated globally. However, failure to achieve polio eradication and competing immunization priorities led to doubts about the political and financial feasibility of eradication. An opportunity to establish a global measles eradication target was missed.

The second decade saw the consolidation of the measles strategy by introducing MCV2, even in settings where MCV1 coverage was low, regular follow-up campaigns, and expansion of the GMRLN. The breakthrough was the 2011 policy decision to link the introduction of RCV to measles elimination efforts and introduce RCV, starting with a broad age range catch-up campaign that would reduce the risk of a paradoxical increase in CRS due to low coverage. Expanding the use of RCV to include low-income countries resulted in a two-thirds reduction in global CRS births by the end of the decade.

As the third decade approached, the sustained success of MRI began to stall. With little further progress toward elimination goals and the absence of new tools or strategies to drive gains, the question was raised by some partners whether the Initiative should be integrated into IA2030, which was championing integrated immunization service delivery. Amid the COVID-19 pandemic, an external assessment of the measles and rubella partnership determined that there were unique functions and essential control strategies that risked being lost if there was no global partnership or common goal.

The first 23 years of the global measles and rubella partnership have generated several lessons learned. The experience has demonstrated the added value of diverse organizations working together toward a shared vision. Dedicated financial and human resources are required for multi-year (2–5 year) program planning. Inter-organizational rivalry and lengthy global policy and strategy discussions can lead to a loss of focus on program performance and country needs, particularly for the most challenging countries. Sustained immunization service delivery requires securing community engagement and demonstrated country ownership, including country ownership of financing, with partners providing supplemental funding rather than sole financing. A common information platform and a sharp focus on regularly tracking performance indicators are needed to identify program weaknesses and allow early course correction. Being responsive to the front-line program needs and “learning from the field” has led to vaccine delivery breakthroughs. While incremental improvements in tactics and best practices will improve program efficiency, new game-changing tools will be needed to change the current trajectory and re-establish momentum [18]. Innovation is urgently needed to address growing anti-vaccine sentiment and the intractable challenge of reaching marginalized communities and vaccinating the more than 20 million infants who miss routine immunization each year. Simpler and safer vaccine delivery technology, such as microarray patches, may help address this inequity and could provide the next leap forward toward regional elimination and eventual eradication of measles and rubella.
